# Books Reviewed

**Published:** 1865-07

**Authors:** 


### BOOKS REVIEWED.

*Annual Report of the City Inspector of the City of New York, for the year ending December* 31, 1864. *Document No.* 7. *New York:* Edmund Jones & Co., 1865.

This work contains the Reports of the various Bureaus of City Inspection. The first, the Bureau of Sanitary Inspection and Street Cleaning—L. H. Boole, Superintendent—refers to the street-cleaning branch of the department, as one of great magnitude, there being 268 miles of paved streets in the city. The whole of this area is swept once every fortnight—about one-quarter is swept three times, and 75 acres twelve times in the same space of time. This Bureau employ 928 men, of various duties, and 290 carts for the daily removal of ashes and garbage.

The duties of the Surveyors of this department are to visit and examine every structure within the city limits. They must ascertain how each is occupied, dimensions of ground, number and size of apartments, height of stories, width of stairways, means of escape in case of fire, and everything pertaining to the public health. The number of occupants of each building must be ascertained. In some of the "tenement houses," measuring 18 feet in width, and 180 feet in depth, 5 stories high, there are 900 persons living. It is not uncommon to notice buildings of ordinary size sheltering 200 or 300 men, women and children. Under this Bureau comes the tables of the returns of ashes, dirt, etc., received at the different dumps.

We notice particularly the elaborate report of Cyrus Ramsay, M. D., L. L. D., Register of Records and Statistics. This report shows that the number of marriages recorded were 2,675; births, 5,877; deaths, 25,645—males, 13,662, females, 11,983. The death-rate was 2.37 per cent., or 22.7 in 1,000. This is the lowest rate since 1851, and less than London or Paris. These returns indicate the city to have been free from any epidemic or endemic, and the general health to have been good.

It furnishes a table, exhibiting the total number of deaths in each year since 1851, which shows that the mortality is decreasing each year; also that there has been but one epidemic in that period, and no endemic of any violence, excepting that scarlet fever and diphtheria prevailed extensively during 1860. Small-pox was more fatal in 1854 and 1861 than usual, typhoid fever during 1863, typhus however declined. It presents tables of the mortality of men, women and children; children under 15 years of age, and under one year of age, &c. Tables of deaths from the most prevalent diseases, and of the number of deaths for each week and month; also tables of comparison between the mortality of New York and other large cities. The report is concluded by some remarks on Sanitary science, &c., and is full and explicit.

---

*Army Surgeon's Manual, for the Use of Medical Officers; from January* 1*st*, 1861, *to April* 1*st*, 1865. By William Grace, *of Washington, D. C. Second Edition.* Bailliere Brothers, 250 Broadway, New York, 1865.

This book contains a detailed List of the Medical Staff of the Army, February 14, 1865, Regulations of the Medical Department, from the Revised Regulations for the Army, General Orders from the Surgeon General's Office, and from the War Department, from 1861 to 1865, and Medical Circulars. To the Army Medical Officer this Manual is invaluable.

*Physicians' Prescription Book; containing lists of the terms, phrases, contractions, and abbreviations used in prescriptions, with explanatory notes; the grammatical construction of prescriptions; rules for the pronunciation of Pharmaceutical terms; a prosodiacal vocabulary of the names of drugs, etc.; and a series of abbreviated prescriptions illustrating the use of the preceding terms. To which is added a Key, containing the Prescriptions in an unabreviated form, with a literal translation, for the use of Medical and Pharmaceutical students.* By JONATHAN PEREIRA, M. D., F. R. S. *Fourteenth edition. Philadelphia:* LINDSAY & BLAKISTON, 1865.

This little manual has reached its fourteenth edition, and speaks for itself. It contains terms and phrases employed in writing prescriptions, nomenclature abbreviations, symbols and signs ancient chemical symbols, grammatical construction, pronunciation of terms, abbreviated and unabbreviated prescriptions, and all that is necessary in writing prescriptions correctly. We heartily recommend every physician to have a copy in his library.

---

*Address before the Medical Society of the State of New York. By* DANIEL P. BISSELL, M. D., *of Utica, President. Delivered February* 3, 1864.

We have to acknowledge the receipt of Dr. Bissell's Address before the Medical Society of the State of New York, on Medical Progress. The Doctor begins as far back in ancient times as Hippocrates, and traces the gradual advance of the science to the present day. The address is eminently entertaining and instructive.

---

*Household Poems.* BY HENRY W. LONGFELLOW. *With Illustrations by John Gilbert, Birket Foster and John Absolon.* Boston: TICKNOR & FIELDS, 1865.

This little volume contains a large number of meritorious and elegant domestic poems, with numerous tasteful illustrations, designed to answer the constant demand for choice, and at the same time, cheap literature. The price of this volume leaves it within the reach of every household.

